# Case Report: A Variety of Immune-Related Adverse Events Triggered by Immune Checkpoint Inhibitors in a Subject With Malignant Melanoma: Destructive Thyroiditis, Aseptic Meningitis and Isolated ACTH Deficiency

**DOI:** 10.3389/fendo.2021.722586

**Published:** 2021-10-12

**Authors:** Yukino Katakura, Tomohiko Kimura, Takashi Kusano, Fuminori Tatsumi, Yuichiro Iwamoto, Junpei Sanada, Yoshiro Fushimi, Masashi Shimoda, Kenji Kohara, Shuhei Nakanishi, Kohei Kaku, Tomoatsu Mune, Hideaki Kaneto

**Affiliations:** Department of Diabetes, Endocrinology and Metabolism, Kawasaki Medical School, Kurashiki, Japan

**Keywords:** immune checkpoint inhibitor, immune-related adverse event, destructive thyroiditis, aseptic meningitis, isolated ACTH deficiency

## Abstract

Recently, immune checkpoint inhibitors have been drawing much attention as cancer immunotherapy, but it has been shown that various immune-related adverse events (irAEs) are induced by immune checkpoint inhibitors in various organs, which has become one of the serious issues at present. A 58-year-old Japanese male with malignant melanoma was treated with nivolumab and/or ipilimumab. During the period of treatment, he suffered from various irAEs. Firstly, about 1 month after starting nivolumab monotherapy, destructive thyroiditis was induced, and so we started replacement therapy with levothyroxine. Secondly, about 1 month after starting nivolumab and ipilimumab combination therapy, aseptic meningitis was induced. We stopped both drugs and started steroid therapy with prednisolone. Finally, about 9 months after restarting nivolumab, isolated adrenocorticotropic hormone (ACTH) deficiency was induced, and so we started replacement therapy with hydrocortisone. Taken together, we should bear in mind the possibility of a variety of irAEs when we use immune checkpoint inhibitors.

## Introduction

Immune checkpoint inhibitors are monoclonal antibodies that exert antitumor effects through the activation of T cells; recently, such inhibitors have been drawing much attention as cancer immunotherapy (1). Monoclonal antibodies against programmed cell death-1 (PD-1) and its ligand PDL-1 and against cytotoxic T-cell antigen-4 (CTLA-4) have been developed, and it has been demonstrated that such antibodies are effective in a variety of malignancies. On the other hand, it has been shown that various immune-related adverse events (irAEs) are induced by immune checkpoint inhibitors in several organs and cells in the whole body, which has become one of the serious issues at present (1). The frequency of endocrine disorders induced by such antibodies is relatively high, and several endocrine organs are damaged in a variety of forms (2). Therefore, we have to perform appropriate therapy for each patient. Here, we show a subject who continuously experienced several irAEs with monotherapy of the anti-PD-1 antibody nivolumab and combination therapy of nivolumab and the anti-CTLA-4 antibody ipilimumab.

## Case Description

**Figure 1:**
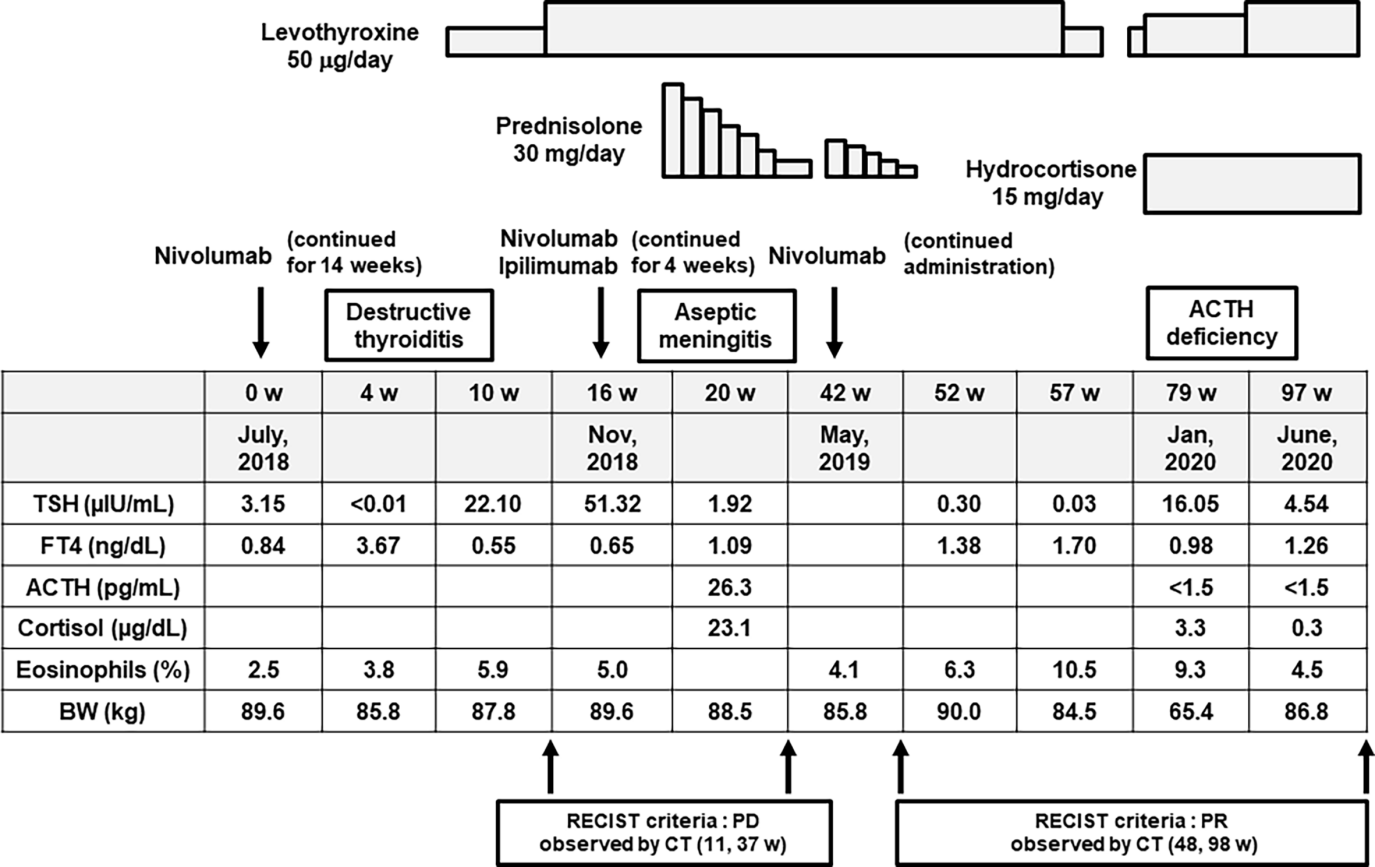
Time course of the clinical parameters, diagnosis, and treatment for this subject. Firstly, about 4 weeks after starting nivolumab monotherapy for malignant melanoma, he suffered from destructive thyroiditis, and so we started replacement therapy with levothyroxine. Secondly, about 4 weeks after starting combination therapy of nivolumab and ipilimumab, he suffered from aseptic meningitis. Thereafter, we stopped both drugs and started steroid therapy with prednisolone. Finally, about 9 months after starting nivolumab, he suffered from isolated adrenocorticotropic hormone (ACTH) deficiency, and so we started replacement therapy with hydrocortisone.

In February, 2018, a 58-year-old Japanese male was diagnosed with malignant melanoma (pT4b, N3, M0, stage IIIC) in the right toe in our dermatology. Tumor resection was performed. He did not have any medical history relevant to this disease and adverse effects. However, since multiple lung and liver metastases were found during the Feron maintenance therapy after the operation, this subject was treated with nivolumab in July 2018 (week 0) (**Figure 1**). The PD-L1 level at the time of treatment was less than 1%, and *BRAF* was negative in this case. About 4 weeks later, thyrotoxicosis was induced [free T4 (FT_4_) = 3.67 ng/dl, thyroid-stimulating hormone (TSH) < 0.01 μU/ml], and about 10 weeks later, hypothyroidism was observed (FT_4_ = 0.55 ng/dl, TSH = 22.10 μU/ml) (**Figure 1**). Therefore, we diagnosed him with nivolumab-induced destructive thyroiditis and started replacement therapy with 50 μg/day of levothyroxine, increasing up to 100 μg/day (**Figure 1**).

In November 2018, nivolumab monotherapy was changed to a combination therapy of nivolumab and ipilimumab in order to enhance the antitumor effect of such medication (**Figure 1**). About 4 weeks later, he had fever and a headache. In the cerebrospinal fluid test, a mononucleosis-dominated cell number increase was observed. Thereby, he was diagnosed with aseptic meningitis induced by the combination therapy of nivolumab and ipilimumab (**Figure 1**). We stopped both drugs and started steroid therapy with 30 mg of prednisolone. We gradually decreased the dose of prednisolone and stopped it in April 2019. Thereafter, we started nivolumab once more because the metastatic tumors were not altered. At that time, we started nivolumab together with prednisolone, just in case, in order to reduce the possibility of recurrence of the adverse effects triggered by nivolumab (**Figure 1**). After stopping prednisolone, however, he felt general fatigue, appetite loss, and nausea, and he had fluid replacement therapy in an outpatient department. Also, the percentage of eosinophils gradually increased up to 10.5% (**Figure 1**).

About 9 months after starting nivolumab, he was hospitalized due to pneumonia. After admission, adrenal insufficiency and hypoglycemia were observed [adrenocorticotropic hormone (ACTH) < 1.5 pg/ml, cortisol = 3.3 μg/dl, postprandial plasma glucose = 64 mg/dl]. Eosinophil was increased to 9.3% [white blood cell (WBC) = 5,390/μl]. In addition, in the rapid ACTH load test, there was no cortisol response. After the diagnosis of adrenal insufficiency, we started replacement therapy with 15 mg of hydrocortisone. After recovery from pneumonia, he was discharged. Since the metastatic tumors in this subject were substantially reduced by the above-mentioned immune checkpoint inhibitors, it seemed that the pathological course of malignant melanoma was relatively favorable, except for the appearance of several adverse effects with the use of such inhibitors.

Thereafter, he was hospitalized again for further examination of adrenal deficiency. On admission, he continued to take 15 mg (10 mg in the morning and 5 mg in the evening) of hydrocortisone and 100 μg of levothyroxine. His height, body weight, and BMI were 168.3 cm, 86.8 kg, and 30.6 kg/m^2^, respectively. The blood pressure, heart rate, and body temperature were 166/99 mmHg, 88bpm, and 36.9°C, respectively. The clinical parameters on admission under replacement therapy with hydrocortisone were as follows: eosinophil, 4.5% (WBC = 7,930/ml); plasma glucose, 95 mg/dl; and HbA1c, 5.5%. Slight hypokalemia was observed (3.3 mmol/L), but renal and liver functions were normal and the lipid parameters were within the normal range. Endocrine system tests at rest revealed low levels of ACTH (<1.5 pg/ml), cortisol (0.3 μg/dl), and dehydroepiandrosterone sulfate (DHEA-S; 6 μg/dl); high levels of luteinizing hormone (LH; 9.77 mIU/ml) and follicle-stimulating hormone (FSH; 26.8 mIU/ml); and normal levels of TSH (4.54 μIU/ml), growth hormone (GH; 0.04 ng/ml), and prolactin (12.1 ng/ml). There was no abnormality in the chest X-ray and electrocardiogram. In sonography, the thyroid size was at the lower limit of normal, and the echo levels were low. In brain computer tomography at the onset of aseptic meningitis, there were no intracranial hemorrhage, space-occupying lesions, or other abnormalities. In contrast-enhanced magnetic resonance imaging (MRI), there were no signs of pituitary swelling, stalk thickness, or space-occupying lesions.

**Figure 2:**
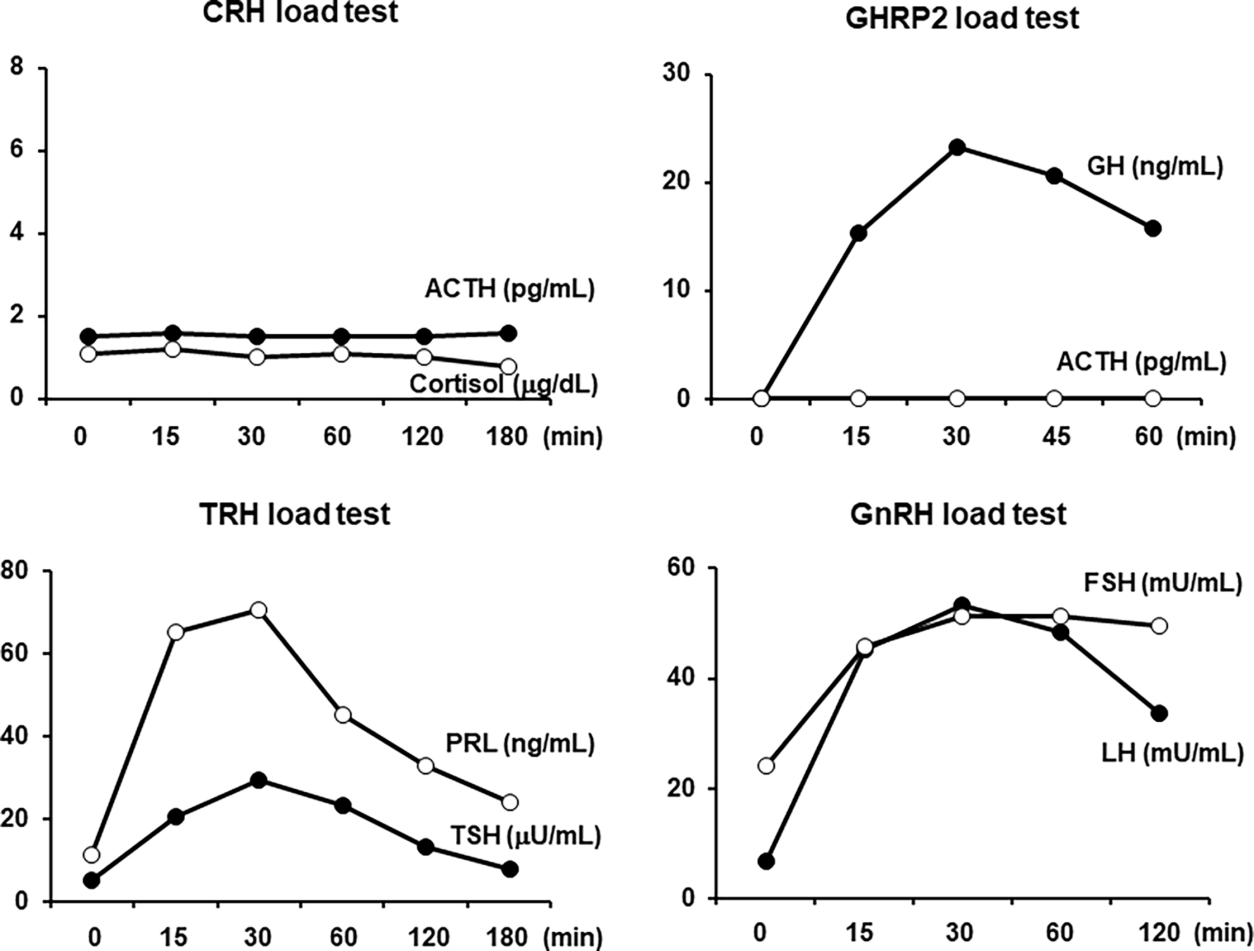
In the corticotropin-releasing hormone (CRH) load test, both the adrenocorticotropic hormone (ACTH) and cortisol levels were not increased at all. In the growth hormone-releasing peptide 2 (GHRP2) load test, the GH level was normally increased, but the ACTH level was not increased at all. In the thyrotropin-releasing hormone (TRH) load test, both the thyroid-stimulating hormone (TSH) and prolactin levels were increased. In the gonadotropin-releasing hormone (GnRH) load test, both the luteinizing hormone (LH) and follicle-stimulating hormone (FSH) levels were normally increased. All load tests were performed in the morning in a fasting state.

All load tests were performed in the morning in a fasting state. As shown in **Figure 2**, in the corticotropin-releasing hormone (CRH) stimulation test, there was no reaction in both the ACTH and cortisol levels, which was compatible with ACTH deficiency. In the GH-releasing peptide 2 (GHRP2) load test, GH showed a normal response, but ACTH did not respond at all, which was also compatible with ACTH deficiency. In addition, as shown in **Figure 2**, in the thyrotropin-releasing hormone (TRH) load test, excess reaction of TSH and normal reaction of prolactin were observed. No increase in FT_3_ secretion was observed in response to TRH. In the gonadotropin-releasing hormone (GnRH) load test, the levels of LH and FSH were normally increased. Based on these findings, we diagnosed this subject with isolated ACTH deficiency and destructive thyroiditis induced by immune checkpoint inhibitors taken for malignant melanoma.

## Discussion

Here, we showed our experience with a subject who had several irAEs with monotherapy of the anti-PD-1 antibody nivolumab and combination therapy of nivolumab and the anti-CTLA-4 antibody ipilimumab. This subject suffered from destructive thyroiditis, aseptic meningitis, and isolated ACTH deficiency, all of which were induced by the use of immune checkpoint inhibitors.

It is known that the anti-PD-1 antibody nivolumab and the anti-CTLA-4 antibody ipilimumab can bring about hypopituitarism, thyroid disorder, adrenal cortex dysfunction, and type 1 diabetes mellitus. Among them, hypothyroidism is mainly induced by anti-PD-1 antibody treatment, and pituitary disorder is mainly induced by anti-CTLA-4 antibody treatment (3). The combination therapy of the anti-PD-1 and anti-CTLA-4 antibodies is expected to show more favorable antitumor effects, but the frequency and the severity of irAEs tend to be higher than those of each agent (4). Indeed, in this subject, aseptic meningitis was induced about 4 weeks after the combination therapy of nivolumab and ipilimumab. Aseptic meningitis has been reported to develop between 1 and 7 weeks after administration of immune checkpoint inhibitors, and it occurs in approximately 0.1%–0.2% of patients treated with ipilimumab (5). Thyroid dysfunction caused by immune checkpoint inhibitors is thought to be mainly destructive thyroiditis, and thyrotoxicosis usually develops as early as 2–6 weeks after the start of administration, followed by hypothyroidism in general. A systematic review reported that the incidences of hypothyroidism and thyrotoxicosis were 3.8% and 1.7% after treatment with an anti-CTLA4 antibody, 7.0% and 3.2% after treatment with an anti-PD-1 antibody, and 13.2% and 8.0% after treatment with anti-CTLA4 and anti-PD-1 antibodies, respectively (3, 6). Indeed, in this subject, about 4 weeks after starting nivolumab, thyrotoxicosis was induced, and 10 weeks later, hypothyroidism was observed.

It is known that the average periods between the start of immune checkpoint inhibitors and the onset of pituitary disorder are about 10 weeks for ipilimumab and several months for nivolumab (7, 8). A systematic review reported that the incidences of pituitary disorders induced by anti-CTLA-4, anti-PD-1, and the combination of anti-CTLA4/anti-PD-1 antibodies were 3.2%, 0.4%, and 6.4%, respectively (3). It is also known that ipilimumab causes multiple anterior pituitary hormone disorders due to hypophysitis (9). In addition, while both nivolumab and ipilimumab can induce isolated ACTH deficiency (8), nivolumab more often causes isolated ACTH deficiency (10, 11). Furthermore, enlargement of the pituitary gland may be observed with ipilimumab administration, but it is rare with nivolumab (8). In our patient, no pituitary swelling was observed. In this case, 3 months after the re-administration of nivolumab (38 weeks after ipilimumab administration), adrenal disorder symptoms appeared at the same time as prednisolone discontinuation. As mentioned above, the MRI scans showed no morphological abnormalities of the pituitary gland. Therefore, this patient was diagnosed with isolated ACTH deficiency due to immune checkpoint inhibitors. From the clinical course, it is probable that the isolated ACTH deficiency in this case developed between the re-administration of nivolumab and the discontinuation of prednisolone, and it was considered that the corticoid effect of prednisolone masked the symptoms of adrenal disorder and delayed the detection. In addition, since there was no evidence that the pharmacological dose of glucocorticoid showed improvements in prognostic effects in pituitary dysfunction triggered by immune checkpoint inhibitors, we used a physiological dose of hydrocortisone. After starting this medication, however, the symptoms promptly disappeared and the subject’s condition stabilized.

In general, the prognosis of malignant melanoma is quite poor, especially when distant metastasis is observed. In this subject, however, it seemed that the pathological course of malignant melanoma was relatively good, except for the appearance of various adverse effects triggered by the aforementioned inhibitors. It was reported that when several side effects were brought about by immune checkpoint inhibitors, the prognosis of the original malignant tumor is relatively good (8, 12, 13), although its precise molecular mechanism remains unknown. Therefore, although speculative, we assume that some modifications in the immune system from the use of the aforementioned immune checkpoint inhibitors contributed to the relatively good prognosis of malignant melanoma in this subject as well. Needless to say, it would be necessary to perform various basic experiments in order to clarify the molecular mechanism how the appearance of adverse effects with the use of such inhibitors leads to exerting some favorable effects on the original malignant tumor.

Taken together, we should bear in mind the possibility of a variety of irAEs, including destructive thyroiditis, aseptic meningitis, and isolated ACTH deficiency, when immune checkpoint inhibitors such as an anti-PD-1 antibody and/or an anti-CTLA-4 antibody are used in subjects with malignant melanoma or other malignant tumors.

## Data Availability Statement

The raw data supporting the conclusions of this article will be made available by the authors, without undue reservation.

## Ethics Statement

Written informed consent was obtained from the individual for the publication of any potentially identifiable images or data included in this article.

## Author Contributions

YK, TKi, TKu, FT, and HK researched data and/or wrote the manuscript. YI, JS, YF, MS, KKo, SN, KKa, and TM contributed to discussion. All authors contributed to the article and approved the submitted version.

## Conflict of Interest

The authors declare that the research was conducted in the absence of any commercial or financial relationships that could be construed as a potential conflict of interest.

## Publisher’s Note

All claims expressed in this article are solely those of the authors and do not necessarily represent those of their affiliated organizations, or those of the publisher, the editors and the reviewers. Any product that may be evaluated in this article, or claim that may be made by its manufacturer, is not guaranteed or endorsed by the publisher.

## References

[1] Kennedy LB Salama AKS. A Review of Cancer Immunotherapy Toxicity. CA Cancer J Clin (2020) 70:86–104. doi: 10.3322/caac.21596 31944278

[2] Chang LS Barroso-Sousa R Tolaney SM Hodi FS Kaiser UB Min L. Endocrine Toxicity of Cancer Immunotherapy Targeting Immune Checkpoints. Endocr Rev (2019) 40:17–65. doi: 10.1210/er.2018-00006 30184160 PMC6270990

[3] Barroso-Sousa R Barry WT Garrido-Castro AC Hodi FS Min L Krop IE. Incidence of Endocrine Dysfunction Following the Use of Different Immune Checkpoint Inhibitor Regimens: A Systematic Review and Meta-Analysis. JAMA Oncol (2018) 4:173–82. doi: 10.1001/jamaoncol.2017.3064 PMC5838579 28973656

[4] Gonzalez-Rodriguez E Rodriguez-Abreu D. Immune Checkpoint Inhibitors: Review and Management of Endocrine Adverse Events. Oncologist (2016) 21:804–16. doi: 10.1634/theoncologist.2015-0509 PMC4943391 27306911

[5] Astaras C de Micheli R Moura B Hundsberger T Hottinger AF. Neurological Adverse Events Associated With Immune Checkpoint Inhibitors: Diagnosis and Management. Curr Neurol Neurosci Rep (2018) 8:3. doi: 10.1016/j.ejca.2016.12.001 29392441

[6] Yamauchi I Sakane Y Fukuda Y Fujii T Taura D Hirata M. Clinical Features of Nivolumab-Induced Thyroiditis: A Case Series Study. Thyroid (2017) 27:894–901. doi: 10.1089/thy.2016.0562 28537531

[7] Caturegli P Di Dalmazi G Lombardi M Grosso F Larman HB Larman T. Hypophysitis Secondary to Cytotoxic T-Lymphocyte-Associated Protein 4 Blockade: Insights Into Pathogenesis From an Autopsy Series. Am J Pathol (2016) 186:3225–35. doi: 10.1016/j.ajpath.2016.08.020 PMC5225294 27750046

[8] Kobayashi T Iwama S Yasuda Y Okada N Okuji T Ito M. Pituitary Dysfunction Induced by Immune Checkpoint Inhibitors Is Associated With Better Overall Survival in Both Malignant Melanoma and Non-Small Cell Lung Carcinoma: A Prospective Study. J Immunother Cancer (2020) 8:e000779. doi: 10.1136/jitc-2020-000779 32606047 PMC7328763

[9] Tsoli M Kaltsas G Angelousi A Alexandraki K Randeva H Kassi E. Managing Ipilimumab-Induced Hypophysitis: Challenges and Current Therapeutic Strategies. Cancer Manag Res (2020) 12:9551–61. doi: 10.2147/CMAR.S224791 PMC7537807 33061641

[10] Gubbi S Hannah-Shmouni F Verbalis JG Koch CA. Hypophysitis: An Update on the Novel Forms, Diagnosis and Management of Disorders of Pituitary Inflammation. Best Pract Res Clin Endocrinol Metab (2019) 33:101371. doi: 10.1016/j.beem.2019.101371 31866206 PMC7078033

[11] Lupi I Brancatella A Cosottini M Viola N Lanzolla G Sgrò D. Clinical Heterogeneity of Hypophysitis Secondary to PD-1/PD-L1 Blockade: Insights From Four Cases. Endocrinol Diabetes Metab Case Rep (2019) 2019:19–0102. doi: 10.1530/EDM-19-0102 PMC6790893 31610523

[12] Freeman-Keller M Kim Y Cronin H Richards A Gibney G Weber JS. Nivolumab in Resected and Unresectable Metastatic Melanoma: Characteristics of Immune-Related Adverse Events and Association With Outcomes. Clin Cancer Res (2016) 22:886–94. doi: 10.1158/1078-0432.CCR-15-1136 PMC4755809 26446948

[13] Osorio JC Ni A Chaft JE Pollina R Kasler MK Stephens D. Antibody-Mediated Thyroid Dysfunction During T-Cell Checkpoint Blockade in Patients With Non-Small-Cell Lung Cancer. Ann Oncol (2017) 28:583–9. doi: 10.1093/annonc/mdw640 PMC5834017 27998967

